# Physiological characteristics and cold tolerance of overwintering eggs in *Gomphocerus sibiricus* L. (Orthoptera: Acrididae)

**DOI:** 10.1002/arch.21846

**Published:** 2021-10-11

**Authors:** Yu Song, Wei‐wei Huang, Yu Zhou, Zhan‐wu Li, Rong Ji, Xiao‐fang Ye

**Affiliations:** ^1^ Xinjiang Key Laboratory of Special Species Conservation and Regulatory Biology, International Center for the Collaborative Management of Cross‐Border Pest in Central Asia, College of Life Sciences Xinjiang Normal University Urumqi China; ^2^ Center for Animal Husbandry and Veterinarians of JiangBei District Chongqing China; ^3^ Monitoring and Reporting Workstation to Prevention and Control of Grasshopper and Mouse of Hami District Hami China

**Keywords:** carbohydrate, cryoprotectants, development, overwintering eggs, supercooling point

## Abstract

*Gomphocerus sibiricus* L., the dominant insect species in the alpine and subalpine grassland, overwinters with diapause at egg stage. In this study, cold tolerance and related cryoprotectants of *G. sibiricus* eggs were investigated. In particular, the supercooling point (SCP), water content, carbohydrates (trehalose, glucose, fructose, glycogen), polyols (glycerol, inositol, sorbitol), fat, and amino acids contents were evaluated at different developmental stages of *G. sibiricus* eggs collected under natural conditions. The SCPs of eggs were very low (−32.83 to −22.61°C) at mid‐diapause. Water content gradually increased during development. The fructose, glycerol, and sorbitol contents were significantly higher in diapausing eggs than in early embryogenesis stage and post‐diapause development stage. Glycogen content was high throughout the whole developmental period. The trehalose, glucose, and inositol contents were low during diapause compared to that in early embryogenesis stage and post‐diapause development stage. There were no significant differences in the fat content of eggs among all development stages. The total amino acid contents in eggs in the early embryogenesis and at the start of diapause were higher than that in post‐diapause eggs. The contents of Glu, Asp, Leu, Pro and Arg during diapause were significantly higher than those during post‐diapause development. Results indicate that *G. sibiricus* eggs have a high supercooling capacity. Successful overwintering can be attributed to the accumulation of glycerol, fructose, sorbitol, and amino acids (Glu, Asp, Leu, Pro and Arg). These findings provide insight into the mechanisms underlying the adaptation of *G. sibiricus* to cold conditions.

## Research Highlights

*Gomphocerus sibiricus* eggs have a high supercooling capacity.

*G. sibiricus* eggs have strong cold resistance.

## Graphical abstract

Physiological characteristics of *Gomphocerus sibiricus* eggs.

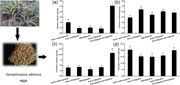

## 1 INTRODUCTION

As an important prerequisite for survival and development, cold tolerance determines the population dynamics and distribution of overwintering insects (Mcdonald et al., 2000). Insects in temperate and frigid zones adjust their physiological state to low temperatures (Bale & Hayward, 2010; Jing & Kang, 2003a; Ren et al., 2016; Zhao et al., 2008). They can behaviorally escape low temperatures by migration or by accessing overwintering habitats. With regard to physiological change, insects can enhance their supercooling capacity and prevent body fluids from freezing by eliminating intracorporal ice nucleation matter, reducing water contents, and accumulating cryoprotectants (Qiang et al., 2008).

The supercooling point (SCP) is a key indicator of cold tolerance in insects (Hao & Kang, 2004; Hodkova & Hodek, 2004). For example, the SCP was used to evaluate the cold hardiness in *Locusta migratoria* (Hao & Kang, 2004; Jing & Kang, 2003b; Li, 2014; Wang & Ablikim, 2006). Freeze‐susceptible insects accumulate cryoprotectants to lower SCPs, while freeze‐tolerant insects elevate SCPs to form protective ice shield (Duman, 1979; Lee, 1989). Furthermore, seasonal variation is an important determinant of the supercooling ability of insects (Yu et al., 2012).

Cold tolerance and diapause are two important factors for the successful overwintering of most insects (Denlinger, 1991). Insects typically resist cold conditions by changing the type or content of cryoprotectants in the body. For example, carbohydrates, fats, polyols, and other cryoprotectants are modulated in their contents during diapause development (Ding & Wu, 2011; Hahn & Denlinger, 2007; Kojić et al., 2018). Therefore, cryoprotectants are regarded as important indicators of insect diapause during the overwintering season at the temperate zone (Gao et al., 2006). Carbohydrates are the main cryoprotectants in insects. For example, in addition to being the main energy source in insects (Arrese & Soulages, 2010), glycogen can be converted into other low molecular weight sugars such as trehalose, fructose and glucose (Storey & Storey, 1991). Polyol is another important indicator of cold tolerance in insects (Ren et al., 2016). It can mediate changes in osmotic pressure caused by dehydration and maintain the structure and stability of proteins, thereby protecting cells from damage caused by low temperatures (Crosthwaite et al., 2011; Kojić et al., 2018).

The accumulation of amino acids contributes to cold tolerance and subsequently for protein synthesis during post‐diapause development (Liu & Wu, 2008). For example, the methyl group of alanine (Ala) is involved in protein shielding in the nucleus (Liang et al., 2003) in addition to the anaerobic metabolic function of Ala itself (Osanai & Yonezawa, 1986). Furthermore, arginine, ornithine, γ‐amino‐n‐butyric acid, cystathionine, and citrulline are related to egg diapause in locusts (Liu & Wu, 2008). An increase in proline is related to the formation of winged insects and tryptophan may be involved in the synthesis of the eye pigment (Liu & Wu, 2008; Reddy & Campbell, 1969).

*Gomphocerus sibiricus* L. is the dominant insect species in the alpine‐subalpine grassland (He et al., 2017; Qiang et al., 2017; Yan et al., 2018). This species gives severe damages to local vegetation along with adverse consequences for animal husbandry. During the egg stage, *G. sibiricus* resists low‐temperature stress. Therefore, in this study, the cold hardiness and physiological protective substances in *G. sibiricus* eggs were investigated during overwintering.

## 2 MATERIALS AND METHODS

### 2.1 Feeding of *G. sibiricus* and collect on of overwintering eggs

During the spawning of *G. sibiricus*, cages were placed in a local grassland (43°22′N, 93°38′E; 2030 m) with collected adults. *G. sibiricus* were fed dominant local plants (*Leymus tianschanicus* and *Stipa capillata*). Nursery pots with soft soil were placed at the bottom of the cage for females to lay eggs. To ensure the same stage of development, eggs were collected regularly every day and then buried under natural conditions for subsequent experiments.

### 2.2 Preparation of *G. sibiricus* eggs at different developmental stages

According to the classification standard of Yan et al. (2018), which combines morphological properties and development time, the development process of *G. sibiricus* eggs were divided into the following five phases. (1) In early embryogenesis (from July to the end of September), Embryos developed rapidly. Compound eyes were basically formed. Antennae and somatic appendages continued to grow. The thoracic and abdominal segments were prominent. (2) At the start of diapause (October), eggs began to enter diapause. Pigment appeared on the edge of the compound eyes. Somatic appendages were clearly segmented. (3) In mid‐diapause (from November to the end of January of the following year), embryos were larger. The appendages and trunk were clearly segmented. The red compound eyes became darker. (4) At the end of diapause (February), compared with the mid‐diapause, morphological changes were not obvious. (5) During post‐diapause development (from early March to the end of April), eggs were released from diapause and embryonic development resumed. Embryos in the egg rotated, and the head turned from the pole direction to the front of the egg. The area of compound eyes increased and the color darkened further. Eggs at different developmental stages were stored in liquid nitrogen tanks for subsequent detection of cold‐resistant substances other than SCP and water content.

### 2.3 Measurements of SCP and environmental soil temperature

**Figure 1:**
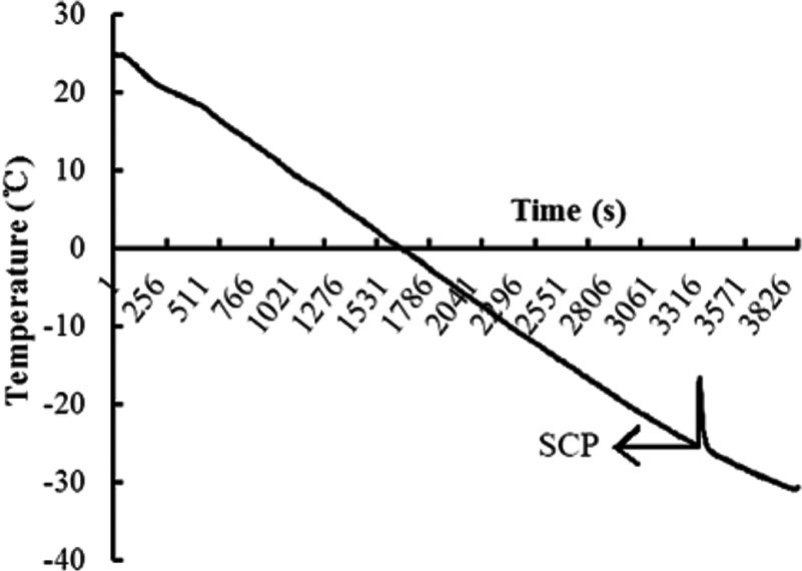
Diagram of the SCP measurements of *G. sibiricus* eggs

SCPs of eggs at different stages were measured. To determine the SCP, each egg was fixed to a thermocouple (with a narrow piece of plastic tape), which was linked to an automatic recorder (TP700; Toprie). Both the thermocouple and the attached egg were protected by a plastic tube and then were cooled to −40°C in a freezing chamber (jw‐2005; Jooway). Cold exposure started at room temperature, resulting in a nonlinear cooling rate of approximately 1°C min^–1^ from 0°C to −40°C. The SCP was indicated on the automatic recorder by a sudden increase in egg temperature. It was represented in the schematic diagram of SCP (Figure 1). The experiment was repeated three times with 20 eggs per group at the same development stage.

Throughout the overwintering process, a geothermometer (TP‐2200, A‐volt) was buried at the location of locust eggs (5 cm below ground) and the temperature was automatically recorded every hour.

### 2.4 Water content in *G. sibiricus* eggs

Eggs at different stages were weighed using an electronic balance (M1) (ME204E/02) and then were placed in an oven at 60°C (DHG9070A, Keelrein) for 72 h until the dry weight (M2) was reached. The water content (%) was calculated as (M1 − M_2_)/ M_1_ × 100. The experiment was repeated three times with 20 eggs per group at the same developmental stage.

### 2.5 Carbohydrate contents in *G. sibiricus* eggs

Eggs at different developmental stages were dried in an oven at 60°C for 24 h and were ground to a powder with a mortar. The glucose content was measured according to the Glucose Determination Kit (Nanjing Jiancheng Institute of Bioengineering). For extraction, the egg powder for each developmental stage was weighed and homogenized fully with 1 mL saline in a homogenizer. The homogenized sample solution was transferred to a centrifuge tube and washed 3 times with 1 mL saline. The washing solution was centrifuged (Centrifuge 5430; Eppendorf) at 3000 *g* for 10 min. The supernatant was obtained for the determination of glucose content following the kit instructions. OD values for samples were measured with a microplate reader (Tecan Infinite 200 Pro) at 505 nm.

The fructose content was measured using a Fructose Determination Kit (Jiangsu Keming Biotechnology Corporation). For extraction, the egg powder for each developmental stage was weighed and mixed well with 1 mL extraction solution in a centrifuge tube. The mixture was heated in a water bath at 80°C for 10 min. The mixed solution was oscillated three to five times, cooled, and centrifuged at 4000 *g* for 10 min. The supernatant was removed. The sediment was mixed well with 1 mL extract solution, centrifuged at 4000 *g* for 10 min, and merged with the supernatant. The fructose content was detected strictly according to the kit instructions. OD values for samples were measured with a microplate reader at 480 nm.

The trehalose content was detected according to the method described by Yi et al. (2009). An appropriate amount of egg powder was weighed and then extracted with 0.5 mL 10% trichloroacetic acid. The mixture was centrifuged at 5000 *g* for 10 min and the supernatant was gently removed. The sediment was repeatedly extracted and the supernatant were merged and volumed to 10 mL. 1 mL sample solution was added into 4 mL anhydrous ethanol for precipitation overnight. After centrifugation, 1 mL supernatant and 2 mL 0.15 mol/L sulfuric acid were added into the tube and heated for 10 min. After cooling, 2 mL 30% potassium hydroxide was added into the tube and mixed well, then heated for 10 min. 1 mL of the above solution and 4 mL 0.2% anthrone solution (0.1 g anthrone + 5 mL 98% sulfuric acid) were mixed in a tube and heated for 10 min. OD values for samples were measured with a microplate reader at 630 nm.

For the detection of the glycogen content, an appropriate amount of egg powder was mixed with 1 mL 30% potassium hydroxide in a 10 mL centrifuge tube and boiled for 30 min. Then, 8 mL of anhydrous ethanol was added, followed by centrifugation at 8100 *g* for 10 min. The supernatant was discarded and the sediment was mixed with 5 mL 0.5 mol/L hydrochloric acid. The mixture was boiled in a water bath for 2 h, filtered, and diluted to 100 mL. 1 mL sample solution and 4 mL 0.2% anthrone solution (0.1 g anthrone + 5 mL 98% sulfuric acid) was added into three test tubes and heated for 10 min after cooling. Then, the tubes were cooled and equilibrated until no bubbles appeared. OD values for samples were measured with a microplate reader at 630 nm.

The glycerol content was detected according to the method described by Wu (2002). The extraction method of the sample solution was the same as that of glucose. 200 µL sample solution was added into a 10 mL centrifuge tube and volumed to 1 mL with distilled water. 2 mL oxidant (130 mg sodium periodate + 8 g anhydrous ammonia acetate + 6 mL glacial acetic acid, then volumed to 100 mL.) was added to each tube and mixed well. Subsequently, 2 mL chromogenic agent (0.4 mL acetylacetone was diluted with isopropanol and volumed to 100 mL) was added into each test tube and mixed well. These tubes were put in a 60°C water bath for 15 min. After cooling, OD values for samples were measured with a microplate reader at 420 nm.

The sorbitol content was detected according to the method described by Wang (2007). The extraction method of the sample solution was the same as that of glucose. 1.2 mL sample solution, 0.18 mL 0.05 g/mL copper sulfate standard solution, and 0.18 mL 0.1 g/mL sodium hydroxide solution were added into three test tubes. The tubes were magnetically stirred for chromogenic reaction for 15 min, and then centrifugated. OD values for samples were measured with a microplate reader at 655 nm.

The inositol content was determined using an insect inositol ELISA Test Kit (Shanghai Jiake Biotechnology Corporation). The extraction method of the sample solution was the same as that of glucose. OD values for samples were measured with a microplate reader at 450 nm.

### 2.6 Fat content in *G. sibiricus* eggs

The fresh weight (FW) of eggs at different developmental stage were weighed and placed in an oven at 60°C for 24 h to obtain the dry weight (DW), and then the egg powder was prepared. Briefly, a 1.5 mL mixture of chloroform and methanol (chloroform: methanol = 2:1, vol/vol) was added and mixed with the egg powder thoroughly in a mortar. Then, 2 mL mixture was centrifuged at 1058 *g* for 10 min and the supernatant was removed. 1.5 mL mixture of chloroform and methanol was mixed with the residue and centrifuged twice. The remaining residue was placed in an oven at 60°C for 72 h to a constant weight (LDW). The experiment was repeated three times with 50 eggs per group at the same development stage.

The content and proportion of fat were calculated by the following formula.

Fatcontent(mg)=DW−LDW.Fatcontentindryweight(%)=[(DW−LDW)/DW]×100.

### 2.7 Amino acid content in *G. sibiricus* eggs

Eggs at different developmental stages were placed in a 60°C oven to dry for 72 h and egg powder was prepared. The obtained egg powder was sent to the Breeding Sheep and Wool Cashmere Quality Safety Supervision and Inspection Centre of the Ministry of Agriculture of China to evaluate the amino acid contents using an automatic amino acid analyzer (S‐433 D; Sykam). The specific analysis methods followed the national standard (GB/T 5009. 124‐2003). The experiment was repeated three times with 30 eggs per group at the same development stage. Amino acid content (%) indicates the percentage of the amino acid in the mass of sample mass.

### 2.8 Statistical analysis

Data were processed and analyzed using single‐factor analysis of variance combined with both LSD and Duncan's tests implemented in Microsoft Excel and SPSS 19.0. The inverse sine square root transformation was required before analyses of percentage data. *p* < 0.05 was defined as significant.

## 3 RESULTS

### 3.1 SCP changes in overwintering eggs

During the entire overwintering period of locust eggs, the SCP decreased initially and increased thereafter, varying from −6.28°C to −32.83°C (Table 1). The SCP of locust eggs did not differ significantly among three development stages (early embryogenesis, start of diapause, and mid‐diapause) (*p* > 0.05). The minimum mean SCP of eggs in mid‐diapause (−30.33°C) was significantly lower than those at the end of diapause and post‐diapause development (*p* < 0.05). The maximum mean SCP of eggs at the end of diapause (−22.46°C) was significantly higher than those at all other stages except for post‐diapause development (*p* > 0.05).

**Table 1:** Changes in the SCPs of overwintering eggs in *G. sibiricus* at different developmental stages

Development stage	Minimum (℃)	Maximum (℃)	Average (℃)	SE	Temperature under −5 cm ground (℃)
Early embryogenesis	−30.8	−6.28	−27.00ab	0.71	17.59
Start of diapause	−32.35	−9.51	−26.81ab	0.59	3.67
Mid‐diapause	−32.83	−22.61	−30.33a	0.13	−1.04
End of diapause	−32.15	−7.26	−22.46c	0.63	−4.23
Post‐diapause development	−31.5	−6.92	−24.32bc	2.4	−1.43

*Note*: Different letters in the same column indicate a significant difference at the 0.05 level.

**Figure 2:**
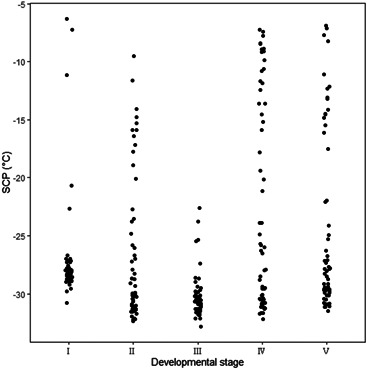
The SCP values in different temperature ranges of locust eggs at different developmental stages. Ⅰ: early embryogenesis; Ⅱ: start of diapause; Ⅲ: mid‐diapause; Ⅳ: end of diapause; Ⅴ: post‐diapause development

The supercooling ability of eggs did not increase significantly with the environmental temperature (Table 1); accordingly, SCP values were further analyzed. As shown in Figure 2, during the entire diapause period, the proportion of eggs with SCP lower than −29.00°C was relatively high. In particular, in mid‐diapause, the proportion of eggs with an SCP lower than −29.00°C was as high as 86.67%, and their values exceeded −17.00°C. In the early embryogenesis stage, the proportion of eggs with SCP values lower than −29.00°C was the lowest (6.90%), but the proportion with values of −28.99 to −23.00°C (84.48%) was higher than that with values of − 22.99 to −5.00°C (8.62%). At the end of diapause and post‐diapause development, the proportions of eggs with an SCP higher than −17.00°C were the highest.

### 3.2 Changes in the water content of overwintering eggs

**Figure 3:**
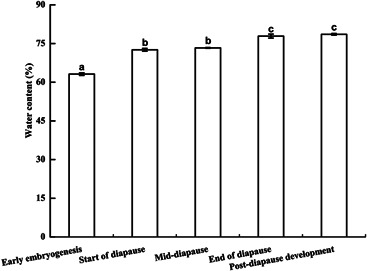
Comparison of the water content of *G. sibiricus* (mean ± *SE*) overwintering eggs at different developmental stages. Each point is an average of three replications. Different letters represent significant differences (*p* < 0.05)

As shown in Figure 3, the water content of overwintering eggs increased gradually during development. It was the lowest in the early embryogenesis, and was significantly lower than those at all other stages (*p* < 0.05). When eggs entered diapause, the water content increased gradually and was significantly higher at the end of diapause than at previous developmental stages (*p* < 0.05). There was no significant difference in water content between post‐diapause development and the end of diapause (*p* > 0.05).

### 3.3 Changes in carbohydrate contents in overwintering eggs

**Figure 4:**
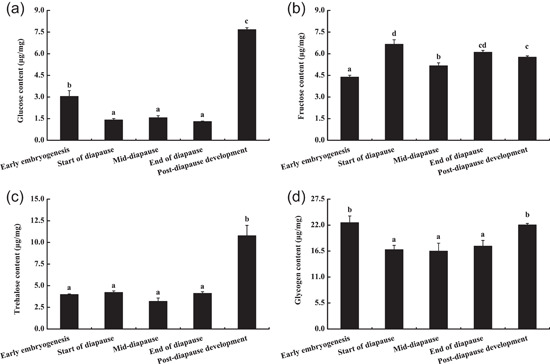
Comparison of the sugars content of *G. sibiricus* (mean ± *SE*) overwintering eggs at different developmental stages. Each point is an average of three replications. For each sugar, mean values followed by different letters are significantly different (*p* < 0.05)

Compared with the other three carbohydrates, the glucose content in eggs was the lowest (Figure 4a), and it showed a U‐shaped trend over time. The glucose content was the lowest in the diapause stage, at which point it was significantly lower than those in the early embryogenesis and post‐diapause development (*p* < 0.05). There were no significant differences in the glucose content during the diapause period (*p* > 0.05). During post‐diapause development, the glucose content (7.67 ± 0.13 μg/mg) was significantly higher than those in all other developmental stages (*p* < 0.05).

The fructose content (Figure 4b) of locust eggs was the lowest in the early embryogenesis (4.39 ± 0.12 μg/mg) (*p* < 0.05). The fructose content at the start of diapause was the highest (6.66 ± 0.31 μg/mg). and was significantly higher than those in all other stages, except the end of diapause (*p* < 0.05). The fructose content decreased significantly during mid‐diapause (*p* < 0.05). It was significantly higher at the end of diapause than in mid‐diapause and the early embryogenesis (*p* < 0.05), but did not differ significantly between the early embryogenesis and post‐diapause development (*p* > 0.05).

The trehalose content (Figure 4c) of locust eggs was low in the early embryogenesis and throughout the diapause period, and with no significant differences among these stages (*p* > 0.05). In the mid‐diapause stage, the trehalose content reached the lowest value (3.20 ± 0.38 μg/mg). In post‐diapause development, the trehalose content (10.77 ± 1.20 μg/mg) was significantly higher than those at all other developmental stages (*p* < 0.05).

The glycogen content remained high throughout the development of locust eggs and showed a U‐shaped trend over time (Figure 4d). The glycogen content of eggs was the highest in the early embryogenesis (22.51 ± 1.43 μg/mg), at which point it was significantly higher than that in the diapause stage (*p* < 0.05). The glycogen content of eggs during diapause was low, with no significant difference among the three stages of diapause (*p* > 0.05). After diapause termination, egg development resumed. The glycogen content increased and was significantly higher than that in the diapause stage (*p* < 0.05).

### 3.4 Changes in the polyol content in overwintering eggs

**Figure 5:**
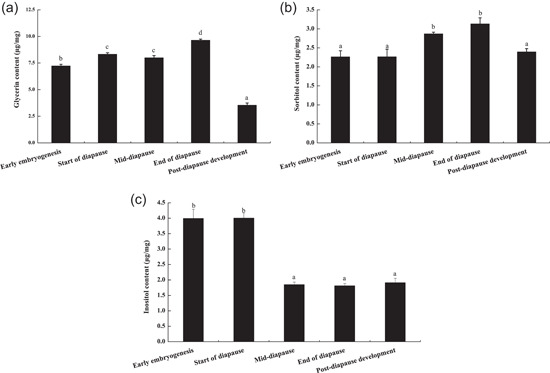
Comparison of the polyols content of *G. sibiricus* (mean ± *SE*) overwintering eggs at different developmental stages. Each point is an average of three replications. For each polyol, mean values followed by different letters are significantly different (*p* < 0.05)

As summarized in Figure 5a, the glycerol content of overwintering locust eggs was relatively high throughout the whole development process and the changing trend was as follows: diapause stage > early embryogenesis > post‐diapause development. The glycerol content in the early embryogenesis was significantly lower than that in the diapause stage, but higher than that in post‐diapause development (*p* < 0.05). After entering diapause, the glycerol content increased significantly (*p* < .05), and was the highest at the end of diapause (9.65 ± 0.11 μg/mg). After the termination of diapause, the glycerol content decreased sharply and was significantly lower than those in all previous developmental stages (*p* < 0.05).

The sorbitol content increased initially and then decreased during the development of locust eggs (Figure 5b). In early embryogenesis, the sorbitol content was the lowest (2.26 ± 0.16 μg/mg), at which point it was significantly lower than those in the mid‐diapause and the end of diapause (*p* < 0.05). As eggs entered diapause, sorbitol accumulated continuously and reached a maximum at the end of diapause (3.13 ± 0.16 μg/mg), at which point it was significantly higher than those in all other stages (*p* < 0.05), except mid‐diapause. After the termination of diapause, the sorbitol content decreased significantly (*p* < 0.05).

The inositol content showed a downward trend during the development of locust eggs (Figure 5c). At the start of diapause, the inositol content (4.01 ± 0.16 μg/mg) was significantly higher than those at all other developmental stages (*p* < 0.05). As eggs continued to develop, the inositol content decreased significantly (*p* < 0.05), with no significant changes thereafter (*p* > 0.05).

### 3.5 Changes in the fat content of overwintering eggs

**Figure 6:**
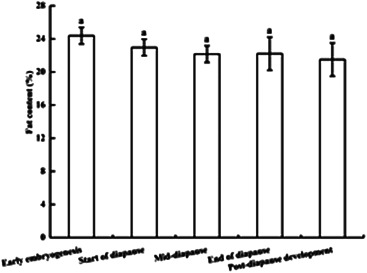
Comparison of the fat content of *G. sibiricus* (mean ± *SE*) overwintering eggs at different developmental stages. Each point is an average of three replications. mean values followed by the same letters are no significant difference (*p* > 0.05)

Throughout the whole developmental process, the fat content of locust eggs showed a decreasing trend. However, there were no significant differences among all developmental stages (*p* > 0.05) (Figure 6).

### 3.6 Changes in the amino acid content of overwintering eggs

During the development of locust eggs, the kinds of amino acids remained unchanged. However, the content of individual amino acids changed (Table 2). The total amount of amino acids was the highest at the start of diapause (58.43 ± 2.25%), at which point it was significantly higher than those in early embryogenesis and in post‐diapause development (*p* < 0.05). In post‐diapause development, the total amount of amino acids in locust eggs (34.88 ± 4.36%) was significantly lower than those at all other stages (*p* < 0.05). Other than these two stages, there were no significant differences in the total amount of amino acids in locust eggs among other developmental stages (*p* > 0.05). Throughout egg development, the contents of Glu, Asp, Leu, Pro and Arg were higher than those of other amino acids, accounting for 16.36%, 8.39%, 7.97%, 6.94%, and 6.87% of the total amino acids, respectively. Except for His, Val, Met, and Cys, the contents of other amino acids during the diapause period were significantly higher than those in post‐diapause development (*p* < 0.05). At the start of diapause, the contents of Glu, Gly, Cys, Val, and Pro in eggs were significantly higher than those the early embryogenesis (*p* < 0.05); in particular, the Cys content increased sharply (75.41%). In mid‐diapause, the contents of all amino acids in locust eggs were lower than those at the start of diapause. The Glu and Val contents decreased significantly by 16.05% and 18.29%, respectively (*p* < 0.05). At the end of diapause, the amino acid content of locust eggs changed slightly but was not significantly different from that in mid‐diapause (*p* < 0.05). In post‐diapause development, except for His, Val, Met, and Cys, the contents of other amino acids were significantly lower than those at the end of diapause (*p* < 0.05), and the contents of most amino acids decreased by more than 20%.

**Table 2:** Changes of amino acid contents (mean ± SE) of *G. sibiricus* overwintering eggs at different developmental Stage

	Changes of amino acid contents of *G. sibiricus* eggs (%)								
Amino acid	Early embryogenesis	Start of diapause	Gradient (%)	Mid‐diapause	Gradient (%)	End of diapause	Gradient (%)	Post‐diapause developmental	Gradient (%)
Asp	4.59 ± 0.27b	4.83 ± 0.17b	5.23%	4.22 ± 0.09b	−12.63%	4.49 ± 0.11b	6.40%	2.91 ± 0.53a	−35.19%
Thr	1.64 ± 0.06ab	2.01 ± 0.13b	22.56%	1.87 ± 0.04b	−6.97%	2.02 ± 0.04b	8.02%	1.35 ± 0.31a	−33.17%
Ser	3.07 ± 0.15ab	3.51 ± 0.20b	14.33%	3.23 ± 0.06b	−7.98%	3.40 ± 0.07b	5.26%	2.31 ± 0.49a	−32.06%
Glu	8.38 ± 0.55b	9.78 ± 0.38c	16.71%	8.21 ± 0.16b	−16.05%	8.83 ± 0.12bc	7.55%	4.62 ± 0.14a	−47.68%
Gly	1.55 ± 0.09ab	2.24 ± 0.11c	44.52%	1.99 ± 0.02bc	−11.16%	2.18 ± 0.07c	9.55%	1.45 ± 0.32a	−33.49%
Ala	3.13 ± 0.15b	3.67 ± 0.12b	17.25%	3.20 ± 0.04b	−12.81%	3.55 ± 0.09b	10.94%	2.43 ± 0.44a	−31.55%
Cys	0.61 ± 0.12a	1.07 ± 0.12b	75.41%	0.74 ± 0.02ab	−30.84%	0.90 ± 0.15ab	21.62%	0.62 ± 0.05a	−31.11%
Val	2.82 ± 0.18a	3.50 ± 0.10b	24.11%	2.86 ± 0.07a	−18.29%	3.17 ± 0.11ab	10.84%	3.01 ± 0.04a	−5.05%
Met	0.87 ± 0.03ab	1.07 ± 0.12b	22.99%	0.89 ± 0.02ab	−16.82%	0.89 ± 0.03ab	0.00%	0.78 ± 0.08a	−12.36%
Ile	2.08 ± 0.16b	2.27 ± 0.11b	9.13%	2.03 ± 0.03b	−10.57%	2.11 ± 0.05b	3.94%	1.50 ± 0.26a	−28.91%
Leu	4.41 ± 0.29b	4.56 ± 0.19b	3.40%	3.96 ± 0.08b	−13.16%	4.19 ± 0.13b	5.81%	2.87 ± 0.63a	−31.50%
Tyr	3.35 ± 0.26b	3.62 ± 0.17b	8.06%	3.15 ± 0.05b	−12.98%	3.29 ± 0.03b	4.44%	2.16 ± 0.44a	−34.35%
Phe	1.82 ± 0.14ab	2.20 ± 0.09b	20.88%	1.92 ± 0.03b	−12.73%	2.10 ± 0.04b	9.38%	1.41 ± 0.28a	−32.86%
His	2.43 ± 0.13a	2.98 ± 0.07a	22.63%	2.50 ± 0.06a	−16.11%	2.72 ± 0.04a	8.80%	2.16 ± 0.52a	−20.59%
Lys	2.37 ± 0.11ab	2.89 ± 0.11b	21.94%	2.62 ± 0.06b	−9.34%	2.83 ± 0.06b	8.02%	1.90 ± 0.45a	−32.86%
Arg	3.47 ± 0.23b	4.04 ± 0.17b	16.43%	3.56 ± 0.07b	−11.88%	3.81 ± 0.11b	7.02%	2.35 ± 0.69a	−38.32%
Pro	2.71 ± 0.12a	4.21 ± 0.18b	55.35%	3.79 ± 0.09b	−9.98%	4.09 ± 0.04b	7.92%	2.59 ± 0.60a	−36.67%
total	49.31 ± 2.98b	58.43 ± 2.25c	18.56%	50.75 ± 0.86bc	‒13.14%	54.57 ± 1.12bc	7.53%	34.88 ± 4.36a	−36.08%

*Note*: Different letters in the same row indicate a significant difference at the 0.05 level.

## 4 DISCUSSION

The cold tolerance of insects during overwintering is crucial for the maintenance of populations, particularly in northern regions where cold tolerance determines the distribution and population size of a species. The cold tolerance of overwintering insects typically changes seasonally, and insects increases gradually as winter approaches and decreases toward the end of winter (Modlmeier et al., 2012; Zheng et al., 2011). *G. sibiricus* mainly occurs in the mountainous area of Hami in Xinjiang. Egg laying mainly occurs in July, adults die in late August, and eggs hatch in May of the next year. Based on changes in environmental temperature and the developmental status, the development of *G. sibiricus* eggs was broadly divided into three main periods: early embryogenesis and the start of diapause (August to October, pre‐winter), mid‐diapause and end of diapause (November to February, winter), and post‐diapause development (March to April, post‐winter).

### 4.1 Changes in SCP egg development in *G. sibiricus*

SCP is a reliable indicator of the cold resistance of locusts (Li, 2014). Greater than 89.6% of *Calliptamus italicus* eggs show an SCP that lower than −24.00°C from September to October (He et al., 2020). The SCPs of *Pararcyplera microptera meridionalis*, *Oedaleus asiaticus*, and *Bryodemella tuberculatum dilutum* that have laid eggs for 90 days were −28.50°C, −27.47°C, and −30.13°C (Li, 2014), respectively. The SCP of overwintering eggs in *Locusta migratoria* reached the minimum value (−23.90°C) in October (Jing & Kang, 2003b). In this study, the SCPs of *G. sibiricus* eggs in the early embryogenesis stage and the start of diapause were low, consistent with the above results for locust eggs. Accordingly, *G. sibiricus* eggs have strong supercooling ability before the winter. In mid‐diapause, the SCP of *G. sibiricus* eggs was the lowest, and up to 98% of locust eggs had SCP values lower than −23.00°C (Figure 2). At this time, the environmental temperature was low and locust eggs were in deep diapause. A low SCP is necessary to resist damage caused by low temperatures (Izadi et al., 2019). At the end of diapause, the environmental soil temperature dropped to the lowest value (−4.23°C), while the average SCP of locust eggs rose to −22.46°C, and the SCP of more than 20% of locust eggs rose to −11.00°C, which may be related to individual development. Although embryonic development is inhibited by low temperatures, locust eggs at the end of diapause may be released from this physiological inhibition in advance and enter the resting period, thus enabling a rapid response to changes in the external environment (Romano et al., 1996). In addition, the soil temperature around the locust eggs was relatively stable and was much higher than air temperature. Combined with the accumulation of cryoprotectants, locust eggs can effectively survive the winter.

In this study, the SCP varied among eggs within a developmental stage. The SCPs of all eggs in the mid‐diapause were lower than −20°C (Figure 2), which was consistent with previous results of migratory locust eggs (Jing & Kang, 2003b). This could be explained by the ability of some individuals to respond quickly to environmental changes, while others require a longer acclimation period, even within a species (Sjursen & Sømme, 2000). The SCPs variation of insects after spring or pre‐winter reflects the acclimation to imminent temperatures change. Under temperature shock, individuals with strong resistance are expected to survive (Chen & Kang, 2005). In contrast, the rapid response of individuals with a change in SCP after spring may expand the ecological range of the population. The lower average SCP of *G. sibiricus* eggs in post‐diapause development than at the end of the diapause stage may be due to the strong‐temperature fluctuations in the spring in Xinjiang, especially the cold temperatures in late spring. Locust eggs respond to the cold stress by decreasing the SCP. The locust eggs in post‐diapause development were released from diapause and developed rapidly. The cold resistance of most eggs decreased as the environmental temperature increased; accordingly, the proportion of locust eggs with an SCP exceeding −17.00°C was relatively high.

The supercooling capacity and response to low‐temperature exposure are commonly used to evaluate the cold tolerance of insects. However, use SCP as a reliable indicator for assessing cold tolerance is a controversial topic (Jing & Kang, 2002). According to SCP and lower lethal temperature, the cold‐tolerance strategies of insects can be divided into three types, including freeze‐tolerant (LT50 < SCP), freeze‐intolerant (LT50 = SCP) and chill‐susceptibility (LT50 > SCP) (Sinclair et al., 2015). Through our repeated attempts, the artificial hatching rate of *G. sibiricus* eggs was low, so the mortality rate after low temperature domestication was not examined in this study. Hao and Kang (2004) found that there was no significant difference in the SCP between pre‐diapause and diapasue eggs in *Chorthippus fallax*, but significantly different from post‐diapause eggs. Furthermore, the SCPs of diapause eggs were close to their LT50. Li (2014) also found that most eggs in *Chorthippus fallax* adopt a freeze‐intolerant strategy to resist low temperature stress. In this study, it was found that the change trend of SCP in *G. sibiricus* eggs was consistent with that of *Chorthippus fallax*. It is speculated that *G. sibiricus* eggs also adapt the freeze‐intolerant strategy to live through the winter, but further studies are needed to be confirmed it.

### 4.2 Changes in small molecule cryoprotectants during egg development in *G. sibiricus*

Cryoprotective agents mainly include trehalose, glucose, fructose, glycerol, sorbitol inositol, and so forth. Some overwintering insects accumulate a kind of substance as the main cryoprotectant agent, while another species accumulate two or more as the cryoprotectants agent system (Bale, 2002; Kojić et al., 2018; Storey & Storey, 1991). Most insects experience some water loss to concentrate body fluids and reduce freezable water when they live through the winter, which may be an important part of cold hardening (Crosthwaite et al., 2011). In this study, the water content of *G. sibiricus* eggs tended to increase, consistent with results of most locust eggs (Chen et al., 2005; He et al., 2020). Previous studies have shown that during overwintering, cold tolerance increases via reductions in the water content or the release of free water from the body to ensure their overwintering survival and population size (Worland, 1996), which is not in agreement with our results. The increase in the water content of *G. sibiricus* eggs, particularly at the start of diapause and at the end of diapause, may be related to the characteristics of locust eggs and the relatively constant soil temperature around eggs, thus decreasing the dependence of cold tolerance on the water content. The specific reasons remain to be further studied.

Carbohydrates are important cryoprotectants for insects (Feng et al., 2016). In this study, the glucose content of eggs at different developmental stages increased in the following order: diapause stage < early embryogenesis < post‐diapause development. It reflects the lack of glucose synthesis during diapause. However, to maintain basic physiological metabolism, a large amount of glucose accumulates in early embryogenesis stage, and is consumed or convert into other substance during diapause. After the termination of diapause, the rapidly developing eggs show enlarged embryos, and large amounts of glucose are synthesized to meet the energy requirements for embryonic development and hatching, explaining the high glucose content. The glucose content in locust eggs was lower than the contents of the other three sugars evaluated in this study. As the primary energy substance, glucose is directly utilized during egg development, so as to consumption of glucose is faster than those of other sugars. The glucose content of *Oedaleus asiaticus* eggs decreases significantly after cold acclimation for 60 days (Li, 2014), which is consistent with our results. On the contrary, the polyol and glucose contents increased in *Gromphadorhina coquereliana* in response to low‐temperature stress (Chowanski et al., 2015). The substantial variation in glucose dynamics in cold resistance of insect may be explained by differences among species, states and habitat of insect.

The fructose content of *G. sibiricus* eggs was the lowest in early embryogenesis and reached a maximum at the start of diapause, thus enabling resistance to low‐temperature stress. It is possible that locust eggs need to consume fructose to maintain diapause, and a portion of fructose may be used to synthesize other cryoprotectants. At the end of diapause, the fructose content increased significantly to prepare for the termination of diapause. In general, the fructose content of *G. sibiricus* eggs during low‐temperature diapause was higher than that during other developmental stages at higher temperatures, consistent with results in other related species. For example, the fructose contents of *Bryodemella tuberculatum dilutum* eggs and *Pararcyplera microptera meridionalis* eggs increased significantly after 30 days of low‐temperature acclimation (Li, 2014); *Cydia pomonella* accumulated a large amount of fructose during the winter (Rozsypal et al., 2013). Our results support the importance of fructose as a cold‐resistant protective substance and an energy source for the development of *G. sibiricus* overwintering eggs.

Trehalose is an important stress‐resistant protective substance in animals and plants. The trehalose content of the *Locusta migratoria manilensis* population in Hainan of China increased after exposure to low temperatures for 24 h (Qi, 2004). *Bryodemella tuberculatum dilutum* eggs can resist cold stress by increasing the trehalose content (Li, 2014). These reports were not inconsistent with our results. We found that the trehalose content of diapause eggs under low environmental temperatures was lower than that of eggs in the early embryogenesis and the post‐diapause development. It indicates that trehalose is not the main cold‐resistant substance in *G. sibiricus* eggs but acts as an energy source. In particular, the accumulation of trehalose in post‐diapause development could provide energy for the smooth hatching of eggs and the subsequent development of nymphs in *G. sibiricus*. Differences among studies may be explained by differences in species and insect states.

Glycogen is a common energy source or protective agent in insects. For example, during the overwintering diapause of *Aspongopus chinensis*, glycogen is important for energy metabolism, and the glycogen and trehalose contents are significantly lower than those after the termination of diapause (*p* < 0.05) (Wu et al., 2019). A portion of larval glycogen in *Dendrolimus spectabilis* larvae is converted to other small carbohydrates during the winter to increase cold tolerance (Han et al., 2005). Glycogen in diapause pupae of *Papilio xuthus* not only serves as an energy reserve but also regulates cold tolerance during the overwintering period (Yi et al., 2009). Glycogen in diapause larvae of *Eurytoma amygdali* is converted to glycol to increase cold tolerance (Khanmohamadi et al., 2016). The cryoprotectants of *Brontispa longissima* adults are trehalose, glycogen, glycerol, and fat (Zhang et al., 2013). In this study, the glycogen content of *G. sibiricus* overwintering eggs was much higher than the glucose, fructose, and trehalose contents, and was maintained at a high level throughout the development period. It indicates that glycogen is the main energy substance during the overwintering period in *G. sibiricus* eggs. It the early embryogenesis, the glycogen content of eggs was the highest. As the environmental temperature drops, the locust eggs entered diapause. To resist the adverse environmental conditions and maintain long‐term diapause, glycogen accumulated in the early embryogenesis may be converted to other sugar alcohol substances. Therefore, glycogen synthesis in eggs mainly occurred in the developmental stage and not during diapause, with particular high synthesis in the early embryogenesis. Although the glycogen content of diapause eggs was low, it was still higher than the contents of other sugars. It suggests that part of the glycogen is stored in the diapause eggs, and the other part is converted into other small carbohydrates for providing energy.

The accumulation of low‐molecular‐weight polyols as cryoprotectants is an important defense measure for insects to resist low‐temperature stress. Glycerol is one of the most important cryoprotectants (Storey & Storey, 1986). The glycerol content of insects increases in response to low temperatures (Zhang et al., 2013). Glycerol, sorbitol, glucose, and trehalose are the main cryoprotectants in overwintering adults of *Hypera postica* under low‐temperature stress (Saeidi & Moharramipour, 2017). The main cryoprotectants in *Anoplophora glabripennis* larvae are glycerol, mannitol, glucose, and amino acids (Feng et al., 2016). Our results showed that the glycerol content of *G. sibiricus* eggs increased initially and then decreased during overwintering. The glycerol content at the diapause stage was significantly higher than that in the early embryogenesis and the post‐diapause development. These findings indicate that the accumulation of glycerol facilitates resistance to cold conditions. In addition, the glycerol content of locust eggs was the highest at the end of diapause, suggesting that the glycerol accumulation provides reserve energy for the release of diapause and subsequent development, thereby ensuring the normal development of locust eggs until hatching. Among the three polyols tested, the glycerol content was the highest, suggesting that glycerol is a major cold‐resistant protective substance for *G. sibiricus* eggs.

Sorbitol protects embryos against cold stress and is important for diapause. For example, the diapause status of *Lymantria dispar* and *Carposina niponensis* eggs could be determined by the sorbitol content (Li et al., 2014; Ren et al., 2016). *Bryodemella tuberculatum dilutum* eggs can increase their supercooling ability by increasing cryoprotectants, such as sorbitol and inositol (Li, 2014). In this study, the sorbitol content of *G. sibiricus* eggs increased first and then decreased during development. These findings indicate that sorbitol is also an important protective substance in *G. sibiricus* eggs, and diapause eggs accumulate sorbitol to survive the winter. At the end of diapause, the sorbitol content of eggs increased significantly and reached the maximum. At the termination of diapause, the sorbitol content decreased significantly. Sorbitol may therefore provide an energy store for egg development after diapause and for rapid development by conversion into sugars. These findings were in agreement with results obtained for *Sitodiplosis mosellana*, which accumulates a large amount of sorbitol during diapause for cold resistance, and sorbitol was converted via sorbitol dehydrogenase into glucose for the termination of diapause (Wang, 2007). It is speculated that sorbitol may be an important indicator to estimate the diapause depth of locust eggs, but which still needs to be verified by further study.

The inositol content under cold conditions varies among different insect species. For example, the accumulation of trehalose and inositol in *Tribolium castaneum* acclimated to low temperatures increases the supercooling capacity (Izadi et al., 2019). After 90 days of low‐temperature acclimation, the inositol content of *Oedaleus asiaticus* eggs increases significantly (Li, 2014). However, the inositol content of *Ostrinia nubilalis* larvae at the middiapause decreases significantly after low‐temperature treatment (Kojić et al., 2018). Similarly, the inositol content of *G. sibiricus* eggs decreased during diapause under low‐temperature conditions. The substantial accumulation of inositol before winter (the early embryogenesis and the start of diapause) helped eggs maintain diapause and resist cold conditions. As the environmental temperature decreased, the locust eggs entered deep diapause and consumed a large amount of inositol that accumulated previously. Therefore, the inositol content was the lowest at the end of diapause. The inositol content increased with the diapause termination of eggs to prepare for hatching. Based on these results, inositol is not the main protective substance of *G. sibiricus* eggs during overwintering. However, it acts as a source for the energy metabolism of eggs to ensure the maintenance of diapause and smooth overwintering.

### 4.3 Changes in the fat content during the development of overwintering eggs

As an energy substance in the growth and development of insects, fat is transformed through a series of metabolic pathways to provide nutrients and energy for biological processes. Overwintering insects use fat as an energy source for survival during the winter. For example, the fat content of diapause pupae in *Papilio xuthus* is much higher than that of non‐diapause pupae (Yi et al., 2009). In this study, although the fat content in *G. sibiricus* eggs tended to decrease during the overwintering period, the observed changes were not significant. The embryos in the early embryogensis are small, and the main component of the egg sheath is the yolk, with abundant fat. During development, embryos gradually become larger and organ development progresses. Despite changes in the locations of fat, the overall content does not change significantly. It suggests that fat is not the main substance related to stress resistance in the diapause stage of overwintering eggs and contributes little to survival the winter. The diapause pupae of *Papilio xuthus* and the overwintering eggs of *G. sibiricus* were in different states. The pupae and eggs respond differently to external conditions, such as temperature and humidity, which may explain the differences in the sources and types of energy. In addition, we have previously found that the main substrates for respiratory metabolism in *G. sibiricus* eggs are lipids before the winter, and lipids and a small amount of sugars during the winter (He et al., 2017). This is consistent with the results of this study showing that the eggs only require small amounts of fat and carbohydrates to meet their respiratory and metabolic needs during the 5‐month diapause period.

### 4.4 Changes in the amino acid content during the development of overwintering eggs

During the overwintering period, the Glu, Asp, Leu, Pro and Arg contents in *G. sibiricus* eggs were high. These five amino acids accumulated substantially before winter and were maintained at high levels during the winter. Therefore, Glu, Asp, Leu, Pro and Arg are likely the main cryoprotectants in *G. sibiricus* eggs during the overwintering period. Glu is the main amino acid that constitutes the protein structure of the developing embryo. In this study, the Glu content was the highest among the amino acids, in agreement with previous findings for *Calliptamus italicus* eggs (Ge et al., 2014). Increases in Asp, Glu, Gly, Ala, Cys, Met and Pro could be indicators of egg diapause (Han et al., 2005; Li, 2014; Liu & Wu, 2008); However, further studies are required to confirm this hypothesis. Most insects produce Ala at low temperatures. The results of this study showed that the Ala content in *G. sibiricus* eggs increased during overwintering. It is possible that in addition to being affected by low temperatures, Ala content can also be increased in early embryogenesis stage by producing energy via anaerobic metabolism (Goto et al., 2001; Liu & Wu, 2008). In post‐diapause development, embryonic development resumed, and both tissues and organs were rapidly constructed in eggs. At this time, the amino acid content decreased continuously due to ongoing consumption to meet the demands of embryonic development and energy metabolism (Table 2).

In summary, *G. sibiricus* eggs under natural conditions showed a strong supercooling ability. The SCPs were lower than the mean temperature of the environment. The water content was closely related to the development of locust eggs. The key cryoprotectants in *G. sibiricus* eggs were glycerol, fructose, sorbitol, and amino acids (Glu, Asp, Leu, Pro and Arg). *G. sibiricus* eggs displayed a strong cold hardiness, suggesting that outbreaks of *G. sibiricus* are possible under the background of climate warming. Owing to the difficulty in artificial breeding and the low artificial hatching rate, only cold resistance of wild *G. sibiricus* eggs under natural conditions was evaluated in this study, while that under artificial incubation conditions was not measured. To elucidate the acclimatized mechanism of *G. sibiricus*, further studies are needed to establish whether cryoprotectants identified in this study can be used as indicators of diapause, to characterize the expression of cold tolerance‐related genes in *G. sibiricus* eggs, and to clarify whether this species is a freeze‐tolerant or freeze‐avoiding insect.

## CONFLICT OF INTERESTS

The authors declare that there are no conflict of interests.

## AUTHOR CONTRIBUTIONS

**Yu Song**: data curation (equal); methodology (equal); resources (equal); writing original draft (equal). **Wei‐wei Huang**: methodology (equal); resources (equal). **Yu Zhou**: investigation (equal); resources (equal). **Zhan‐wu Li**: investigation (equal); resources (equal). **Rong Ji**: resources (equal); supervision (equal). **Xiao‐fang Ye**: conceptualization (equal); data curation (equal); methodology (equal); project administration (equal); supervision (equal); writing review & editing (equal).

## Data Availability

The data that support the findings of this study are available from the corresponding author upon reasonable request.
